# Dysregulated miRNAome and Proteome of PPRV Infected Goat PBMCs Reveal a Coordinated Immune Response

**DOI:** 10.3389/fimmu.2018.02631

**Published:** 2018-11-21

**Authors:** Alok Khanduri, Amit Ranjan Sahu, Sajad Ahmad Wani, Raja Ishaq Nabi Khan, Aruna Pandey, Shikha Saxena, Waseem Akram Malla, Piyali Mondal, Kaushal Kishor Rajak, D. Muthuchelvan, Bina Mishra, Aditya P. Sahoo, Yash Pal Singh, Raj Kumar Singh, Ravi Kumar Gandham, Bishnu Prasad Mishra

**Affiliations:** ^1^Division of Veterinary Biotechnology, ICAR-Indian Veterinary Research Institute (IVRI), Bareilly, India; ^2^DBT-National Institute of Animal Biotechnology, Hyderabad, India; ^3^The Ohio State University, Columbus, Ohio, OH, United States; ^4^Division of Biological Products, ICAR-Indian Veterinary Research Institute (IVRI), Bareilly, India; ^5^Division of Virology, ICAR-Indian Veterinary Research Institute (IVRI), Mukteswar, India; ^6^ICAR- Directorate of Foot and Mouth Disease, Mukteswar, India; ^7^ARIS Cell, ICAR-Indian Veterinary Research Institute (IVRI), Bareilly, India

**Keywords:** miRNAome, proteome, PPR, goats, host-pathogen interaction, immunopathogenesis

## Abstract

In this study, the miRNAome and proteome of virulent Peste des petits ruminants virus (PPRV) infected goat peripheral blood mononuclear cells (PBMCs) were analyzed. The identified differentially expressed miRNAs (DEmiRNAs) were found to govern genes that modulate immune response based on the proteome data. The top 10 significantly enriched immune response processes were found to be governed by 98 genes. The top 10 DEmiRNAs governing these 98 genes were identified based on the number of genes governed by them. Out of these 10 DEmiRNAs, 7 were upregulated, and 3 were downregulated. These include miR-664, miR-2311, miR-2897, miR-484, miR-2440, miR-3533, miR-574, miR-210, miR-21-5p, and miR-30. miR-664 and miR-484 with proviral and antiviral activities, respectively, were upregulated in PPRV infected PBMCs. miR-210 that inhibits apoptosis was downregulated. miR-21-5p that decreases the sensitivity of cells to the antiviral activity of IFNs and miR-30b that inhibits antigen processing and presentation by primary macrophages were downregulated, indicative of a strong host response to PPRV infection. miR-21-5p was found to be inhibited on IPA upstream regulatory analysis of RNA-sequencing data. This miRNA that was also highly downregulated and was found to govern 16 immune response genes in the proteome data was selected for functional validation vis-a-vis *TGFBR2* (TGF-beta receptor type-2). *TGFBR2* that regulates cell differentiation and is involved in several immune response pathways was found to be governed by most of the identified immune modulating DEmiRNAs. The decreased luciferase activity in Dual Luciferase Reporter Assay indicated specific binding of miR-21-5p and miR-484 to their target thus establishing specific binding of the miRNAs to their targets.This is the first report on the miRNAome and proteome of virulent PPRV infected goat PBMCs.

## Introduction

MicroRNAs (miRNAs) are small non-coding RNAs (22 nucleotides) found to regulate the expression of genes post-transcriptionally in animals, plants, and some viruses (1). They regulate different cellular processes, including reproduction, development, pathogenesis, and apoptosis (2–4). miRNAs are also effective in regulating immune response and cellular differentiation (5–7). The regulation process generally takes place by binding of miRNA at its seed sequence (2–8 nucleotides from 5′-end) to the 3′ untranslated region (3′UTR) of specific mRNAs of the genes that govern the biological processes. However, several instances of miRNAs binding to 5' UTR or coding regions in the regulation process have also been reported (8, 9).

Viral pathogenesis is greatly influenced by cellular miRNAs (10–12). Several cellular miRNAs have been demonstrated to play a regulatory role in the host-virus interaction networks (13, 14). Cellular miRNA expression profile is profoundly influenced by viral infections and vice-versa (15). For example, miR-122 is reported to enhance replication of Hepatitis C virus (16) and miR-142 suppresses replication of Eastern Equine Encephalitis virus (17). The HIV-1 virus has been found to increase the expression of various host miRNAs, including miR-370, miR-122, miR-297, and miR-373, and suppress the expression of miR-17-92 cluster (18). With the advent of deep sequencing technology, it has become possible to explore changes in miRNA expression in the host, in response to various viral infections like enterovirus 71, avian influenza, PPRV, Japanese Encephalitis virus and hepatitis C virus (19–23).

Peste des petits ruminants (PPR) characterized by fever, sore mouth, conjunctivitis, gastroenteritis, and pneumonia, is an acute, highly contagious viral disease of sheep and goats. However, a more severe form of the clinical disease has been reported in goats than in sheep, since goats are more susceptible (24–29). The recovery is also slower in goats than in sheep (27). However, regions having large sheep populations have reported severe outbreaks of PPR (27, 30, 31). Our earlier *in-vitro* transcriptome analysis studies to evaluate host response of goat PBMCs to PPR live attenuated vaccine virus uncovered several transcription factors that modulate immune response (32, 33). Also, dysregulation in the host miRNAome in lung and spleen of experimentally infected goats and sheep by virulent PPRV suggests a strong host immune response in sheep and goats (21). However, the host miRNAome of PPRV infected goat and sheep PBMCs has not been explored to date. Lymphocytes are the primary targets of PPRV infection from where it reaches different tissues by piggybacking on PBMCs (34). A higher viral load is reported to be at 9 days post-infection (dpi), which coincides with the peak clinical signs of the disease (22, 35). In the present study, control (0 day) and PPRV infected PBMCs (9 dpi) of goats were isolated and subjected to microRNA sequencing (miRNA-seq) and proteome profiling. DEmiRNAs were identified from miRNA-seq data and correlated with the proteome data to identify the miRNAs that govern the immune processes. Among the miRNAs, miR-21-5p was found to be highly downregulated in miRNA-seq data, inhibited in RNA sequencing (RNA-seq) data (unpublished) and involved in regulation of various immune response genes. This miR-21-5p was selected for annotation and functional validation. Additionally, one more miR, miR-484 was randomly chosen from top 10 immunoregulating DEmiRNAs for functional validation.

## Materials and methods

### Ethics statement and animal experiment

The study is a part of vaccine potency testing experiment conducted at ICAR-Indian Veterinary Research Institute Mukteshwar Campus as per the guidelines of Indian pharmacopeia-2014. The permission to conduct the experiment was sought from Indian Veterinary Research Institute–Institutional Animal Ethics Committee (IVRI–IAEC) under the Committee for the Purpose of Control and Supervision of Experiments on Animals (CPCSEA), India and was approved vide letter no 387/CPCSEA. The animals that were apparently healthy and negative for the presence of PPRV antibody by competitive ELISA and serum neutralization test (SNT) were used in this study. Virulent PPRV [accession number KR140086.1 (36)], a lineage IV isolate and strain Izatnagar/94, was used as a challenge virus and infection was confirmed in goats by RT–PCR, qRT-PCR, and sandwich ELISA. PBMCs were isolated from the blood collected from PPRV (Izatnagar/94) infected goats at 9 dpi (The animals succumbed to the disease at 10 dpi). The PBMCs isolated from blood collected from apparently healthy animals (0 day) acted as a control. PBMCs were isolated using Histopaque-1077 (Sigma), USA.

### MicroRNA sequencing

Total RNA from the PBMCs of goats was isolated using the RNeasy Mini kit (Qiagen GmbH, Germany) following the manufacturer's protocol. To access integrity and quality of the RNA, RNA integrity number (RIN) value of each sample was measured on Bioanalyzer (Agilient Technologies, Inc.). The RIN value was found to be >8, which is considered suitable for further processing (37). The library was prepared using NEBNext Multiplex Small RNA Library Prep Kit (New England Biolabs Inc.) as per the manufacturer's protocol. Hundred nanogram of total RNA from each sample was used for small RNA library preparation. The quality of the libraries was assessed on Bioanalyzer. Libraries were quantified using Qubit 2.0 Fluorometer (Life Technologies) and by qPCR (38).The high-throughput sequencing was performed on Illumina–NextSeq500 platform to generate 75 bp single-end reads as per manufacturer's protocol. The data was submitted to the GEO database with accession number GSE109799.

### Processing miRNA-seq data

The miRNA reads trimming and preprocessing was performed with CLC Genomic workbench v6.0 (CLC bio, Denmark) to remove adaptor sequences and low quality reads using default parameters. Since the cattle genome (mirBase–Release 21) is relatively better annotated and as the miRNAs are conserved across species, cattle genome was used to map these clean reads. The map files for the infected and control samples were created independently. From the toolbox of CLC workbench, an experiment was created, the read count was quantitatively normalized and the expression values were obtained. Proportion based statistics–Kal's test was used to identify differentially expressed miRNAs at 9dpi PPRV infected PBMCs of goat.

### Proteomics data generation and analysis

The proteomic data was generated from control and PPRV infected 9 dpi goats PBMCs and analyzed following the standard procedure as described in the previous study (21). Briefly, proteome from goat PBMCs was quantitatively analyzed using trypsin in conjugation with C18 Nano-LC column separation, followed by analysis on the Waters Synapt G2 Q-TOF instrument for MS. The raw data was processed by MassLynx 4.1 WATERS, and MSMS spectra were matched to the database sequence using PLGS software. The identified proteins in the three runs of each sample were compared with each other as control (healthy) and infected samples. Quantification was done using expression analysis package of the PLGS software. The ion counts matching with the peptides of a specific protein corresponding between the two samples in the three runs were averaged and the ratio was calculated for the whole protein.

### Functional annotation of differentially expressed miRNAs

To explore the regulatory role of the differentially expressed miRNAs, the target genes governed by each of the DEmiRNAs were obtained using TargetScan tool (39)^1^ All these target genes were pooled up and compared to the dysregulated proteins identified in proteomics data. The genes common to both data were selected for functional annotation via ClueGo (ver. 2.3.3) and CluePedia (ver. 1.3.3) (40) in Cytoscape (ver. 3.2.1) (41). The genes involved in top 10 significantly enriched immune response processes were identified. Out of these immune response genes, the genes governed by each of the DEmiRNAs were identified and top 10 DEmiRNAs were selected.

### Processing RNA-seq data

**Figure 1:**
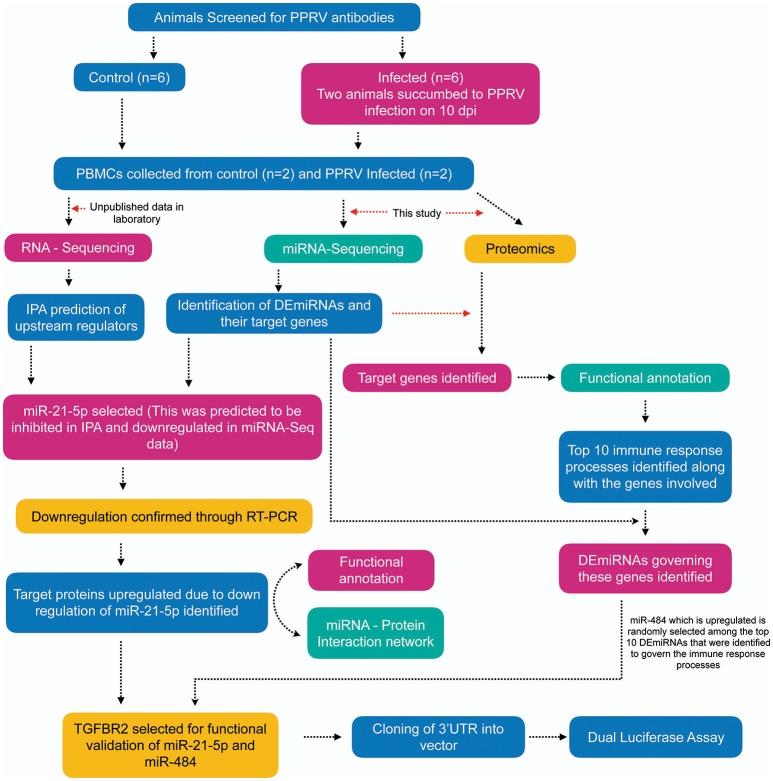
Overview of the experiment. Apparently healthy goats (*n* = 6) that were negative for the presence of PPRV antibody by competitive ELISA and serum neutralization test (SNT) were infected with virulent PPRV strain Izatnagar/94. These animals acted as control in the vaccine potency experiment. Two animals in the group succumbed to disease on 10 dpi. PBMCs from blood collected at 9dpi from these two succumbed animals and PBMCs isolated from blood collected on 0 day (control) were sent for miRNA-Sequencing, RNA-Sequencing (unpublished data in the lab) and Proteome profiling. Differentially expressed miRNAs (DEmiRNAs) and proteins were identified from the miRNA-Seq and proteome data, repectively. The DEmiRNAs were functionally annotated w.r.t the proteins governed in the proteome data. The top 10 DEmiRNAs governing the top 10 immune response processes were identified. miR-21-5p which was found to be inhibited on IPA upstream regulatory analysis of RNA-Sequencing data, highly downregulated in miRNA-Seq data (validated by qRT-PCR) and found to govern 16 immune response genes in the proteome data was selected for functional validation vis-a-vis *TGFBR2* (TGF-beta receptor type-2). *TGFBR2* regulates cell differentiation and is involved in several immune response pathways was found to be governed by most of the identified immune modulating DEmiRNAs. Dual luciferease assay was done using mimics of miR-21-5p and miR-484 and 3′UTR of the target gene *TGFBR2* to establish the specificity of binding.

From IPA analysis (Ingenuity Pathway Analysis)^2^ of the RNA-sequencing data generated in our lab (unpublished), the upstream miRNA regulators governing the differentially expressed genes (data not shown) were identified. The overview of the entire analysis is given in Figure 1.

### Downregulation of miR-21-5p in qRT-PCR

The expression of the miR-21-5p in PPRV infected PBMCs was validated by qRT-PCR. Total RNA, including small RNA from the PBMCs of control and infected goats, was isolated using mirVana™ miRNA isolation kit (Invitrogen). Reverse transcriptase reactions were performed using RT specific primers of miR-21-5p and U6snRNA by TaqMan® MicroRNA Reverse Transcription Kit (Applied Biosystems, USA, #4366596). Total RNA from the PBMCs was diluted to a concentration of 10 ng/μl and 1 μl of RNA was added to the reaction mix containing 0.15 μl 100 mM dNTPs, 1 μl of RT enzyme (50 U/μl), 1.5 μl 10 × RT buffer, 0.19 μl RNase inhibitor (20 U/μl), 3 μl 5 × RT specific-primer and 8.16 μl DEPC-treated water to obtain a final volume of 15 μl. The reaction conditions were 16°C for 30 min followed by 42°C at 30 min and 85°C for 5 min to stop the reaction. The cDNA was then used for the Real-time PCR. Real-time PCR was performed using a standard TaqMan PCR kit protocol on Applied Biosystems 7,500 fast Sequence Detection System. The 10 μl PCR included 5 μl of 2 × Taqman Gene Expression Mastermix (Thermo Fisher Scientific Inc., Wilmington, DE, USA), 0.5 ul of 20 × Taqman probe (Assay ID 005982-mat), 2 μl (0.134 ng) of RT product and 2.5 μl of NFW. The reactions were incubated in a 96-well plate at 95°C for 10 min, followed by 40 cycles of 95°C for 15 s and 60°C for 1 min. All reactions were run in triplicate. The expression of miRNA-21-5p was assayed taking the expression of U6snRNA as an internal control. The relative expression of miR-21-5p was calculated using the 2^−ΔΔCT^ method with the control group as calibrator (42). Student's *t*-test was done in JMP9 (SAS Institute Inc., Cary, USA) to test the significance of difference and difference between groups was considered significant at *P* ≤ 0.05.

### Prediction of miR-21-5p target genes and functional annotation

The target genes governed by miRNA-21-5p were obtained using TargetScan tool to explore its regulatory role (39). The target genes obtained were compared to the upregulated proteins from proteome data to identify the proteins that are upregulated because of downregulation of miR-21-5p. The miRNA-protein-network was created based on the expression profile of target genes and miR-21-5p using Cytoscape (ver. 3.2.1). Functional annotation of these genes was performed by ClueGo (ver. 2.3.3) and CluePedia (ver. 1.3.3) (40) in Cytoscape (ver. 3.2.1) (41).

### Functional validation of miR-21-5p and miR-484

#### Prediction of miR target site in 3′ UTR

*TGFBR2* that is governed by miR-21-5p and miR-484, and found connected to most significantly enriched GO terms, was selected for further validation of the miRNAs. The 3'UTR target site of this gene and mature miRNA were extracted from NCBI and analyzed in miRanda (43) tool to evaluate the strength of interaction using the parameters ΔG and total score value.

#### Design of wild type and mutant type miR target sites and miRNA mimic and control

While two wild-type oligonucleotides (62 bp) were constructed from 3′UTR of *TGFBR2* mRNA flanking the miR-21-5p and miR-484 target sites, respectively, the mutant of both was created by replacing the target site either with poly A or poly T sequence. pGL4.13 vector (Promega) was used to clone the oligonucleotide sequences (wild-type and mutant-type separately) at XbaI RE site of this vector. pGL4.74 was used as a control vector for the normalization of the transfection efficiency. Likewise, mimics of miR-21-5p and miR-484 were chemically synthesized. miR-67-3p was used as control for it is reported to have least sequence identity with known miRNAs in humans, rat, and mouse (44).

#### Co-transfection strategy for carrying out dual-luciferase reporter assay

HEK293 cells were used for the co-transfection of the vectors, pGL4.13, and pGL4.74. The cells were maintained in MEM medium with 10% FBS, antibiotic and antimycotic (Himedia), and placed in an incubator at 37°C with 5% CO_2_. The experiment was performed in triplicates. Briefly, the co-transfection complex for each vector was prepared in 2 tubes, one containing Opti-MEM + Lipofectamine and the other tube containing Opti-MEM + Vectors + Mimic. The complex was formulated as shown in Table 1. Contents of tube A and B were mixed and incubated for 15 min at room temperature to allow the formation of transfection complex. After the formation of the complex, Opti-MEM was added to the complex to make the volume up to 400 μl. The medium from the 24 well plate was removed and the co-transfection complexes were gently loaded into each well of the plate. The plate was kept in an incubator for 4 h. After 4 h, the medium with the complex was removed from the wells and fresh 1% MEM (500 μl) was added to the wells. The cells were lysed 48 h post co-transfection and the luciferase activity was measured using Dual Luciferase Assay kit (Promega) according to the manufacturer's protocol. The assay results were represented as relative luciferase activity. Student's *t*-test was done in JMP9 (SAS Institute Inc, Cary, USA) to test the significance of difference, and differences between groups were considered significant at *P* ≤ 0.05.

**Table 1:** Co-transfection complex for the target gene against miRNAs.

	**Well 1**	**Well 2**	**Well 3**	**Well 4**
**Tube A**	TGFBR2 Wild (1 μg)	TGFBR2 Wild (1 μg)	TGFBR2 Mutant (1 μg)	TGFBR2 Mutant (1 μg)
	pGL4.74 (5 ng)	pGL4.74 (5 ng)	pGL4.74 (5 ng)	pGL4.74 (5 ng)
	miR-21-5p/484 mimic (25 μM)	miR-67-3p (25 μM)	miR-21-5p/484 mimic (25 μM)	miR-67-3p (25 μM)
	P3000 Reagent (1 μl)	P3000 Reagent (1 μl)	P3000 Reagent (1 μl)	P3000 Reagent (1 μl)
	Opti-MEM (50 μl)	Opti-MEM (50 μl)	Opti-MEM (50 μl)	Opti-MEM (50 μl)
**Tube B**	Lipofectamine3000 (1.5 μl)	Lipofectamine3000 (1.5 μl)	Lipofectamine3000 (1.5 μl)	Lipofectamine3000 (1.5 μl)
	Opti-MEM (50 μl)	Opti-MEM (50 μl)	Opti-MEM (50 μl)	Opti-MEM (50 μl)

## Results

### Confirmation of PPRV infection

**Figure 2:**
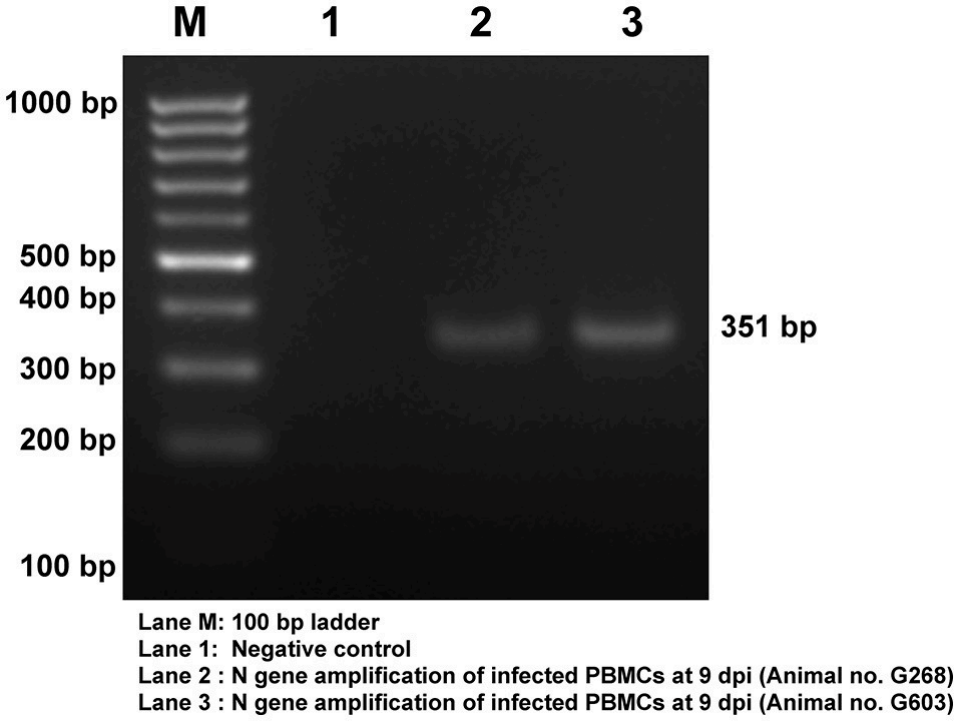
Confirmation of PPRV infection in PBMCs of goats. Amplification of 351 bp N gene by RT- PCR. Lane M, 100 bp ladder; Lane 1, NTC; Lane 2, Infected Goat PBMCs (Animal no: G268) Lane 3, Infected Goat PBMCs (Animal No: G603).

Viral infection in the PBMCs of goats infected with PPRV was confirmed by RT-PCR amplification of 351 bp N gene fragment (Figure 2). The viral infection was further confirmed by sandwich ELISA and qRT-PCR (data not shown).

### miRNAs governing the immune processes were identified

**Figure 3:**
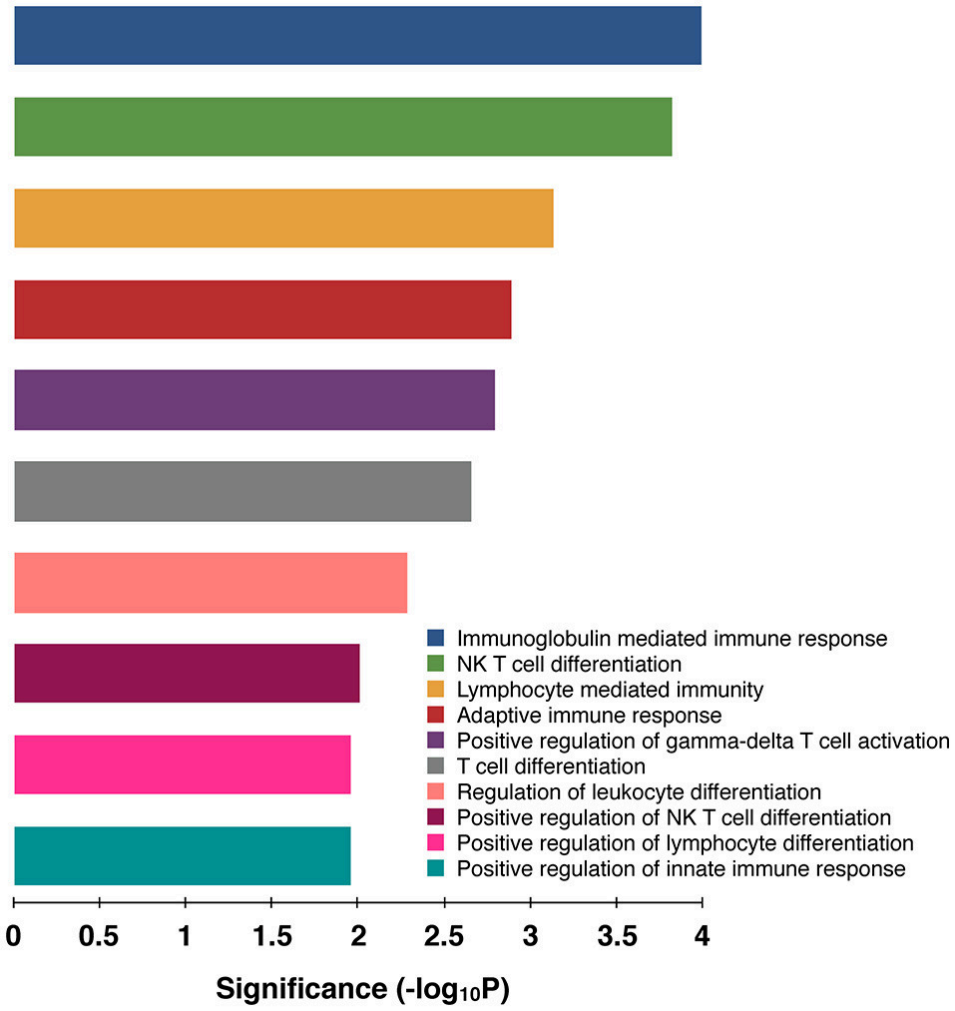
Top 10 Significant (*P* < 0.05) Immunological processes involved in virulent PPRV goats PBMCs. Color differentiates the immune processes.

In goat PBMCs infected with PPRV, a total of 68 miRNAs were significantly (*P* < 0.05) differentially expressed (42 down-regulated and 26 up-regulated) (Table S1). From the proteomics data (Table S2) generated from PPRV infected goat PBMCs, 1,965 and 3,509 proteins were identified to be downregulated and upregulated, respectively. From the TargetScan data, 15,341 genes were found to be governed by the 68 DEmiRNAs, out of which 4,027 proteins were found to be dysregulated in the proteomics data. On ClueGo analysis of these genes, the top 10 significantly enriched immunological processes included immunoglobulin mediated immune response, NK T cell differentiation lymphocyte mediated immunity, adaptive immune response, positive regulation of gamma-delta T cell activation, T cell differentiation, regulation of leukocyte differentiation, positive regulation of NK T cell differentiation, positive regulation of lymphocyte differentiation, positive regulation of innate immune response (Figure 3). The genes (from the proteomics data) enriched under these GO terms are given in Table S3. A total of 98 genes were found to be enriched in these top 10 significant immune response processes governed by 42 DEmiRNAs (18 upregulated and 24 downregulated) (Tables S4, S5). The top 10 DEmiRNAs based on the number of immune response genes governed are given in Table 2. On comparing the DEmiRNAs of PPRV infected PBMCs with DEmiRNAs of the PPRV infected lung and spleen, reported in our earlier study (21), we found there are 3 DEmiRNAs common among the lung, spleen and PBMCs and 9 DEmiRNAs common between the lung and PBMCs. However, there was no DEmiRNA exclusively common between PBMCs and spleen (Table S6). Of the 9 DEmiRNAs common between lung and PBMCs, miR-378b, miR-342, miR-30f, miR-339a were found to be downregulated while miR-1246 and miR-2440 were upregulated in both. In addition, miR-181a-1, miR-181a-2 and miR-7-1 were found upregulated in lung but downregulated in PBMCs. Of the 3 DEmiRNAs common to each, miR-574 was found upregulated. miR-21-5p was found upregulated in spleen and lung but downregulated in PBMCs and vice-versa in case of miR-744.

**Table 2:** Ten DEmiRNAs based on the number of immune response genes, involvement of *TGFBR2* and fold-change values.

	**miRNAs**	**No. of Immune response genes**	***TGFBR2***	**log_2_FC**
1	miR-664	33	Yes	1.37
2	miR-2311	31	Yes	1.15
3	miR-2897	27	-	1.85
4	miR-484	27	Yes	1.14
5	miR-2440	22	Yes	0.13
6	miR-3533	19	-	1.87
7	miR-574	18	Yes	0.83
8	miR-210	17	Yes	**–**2.70
9	miR-21-5p	16	Yes	**–**7.20
10	miR-30	15	-	**–**6.97

### miR-21-5p was selected for functional annotation and validation

**Figure 4:**
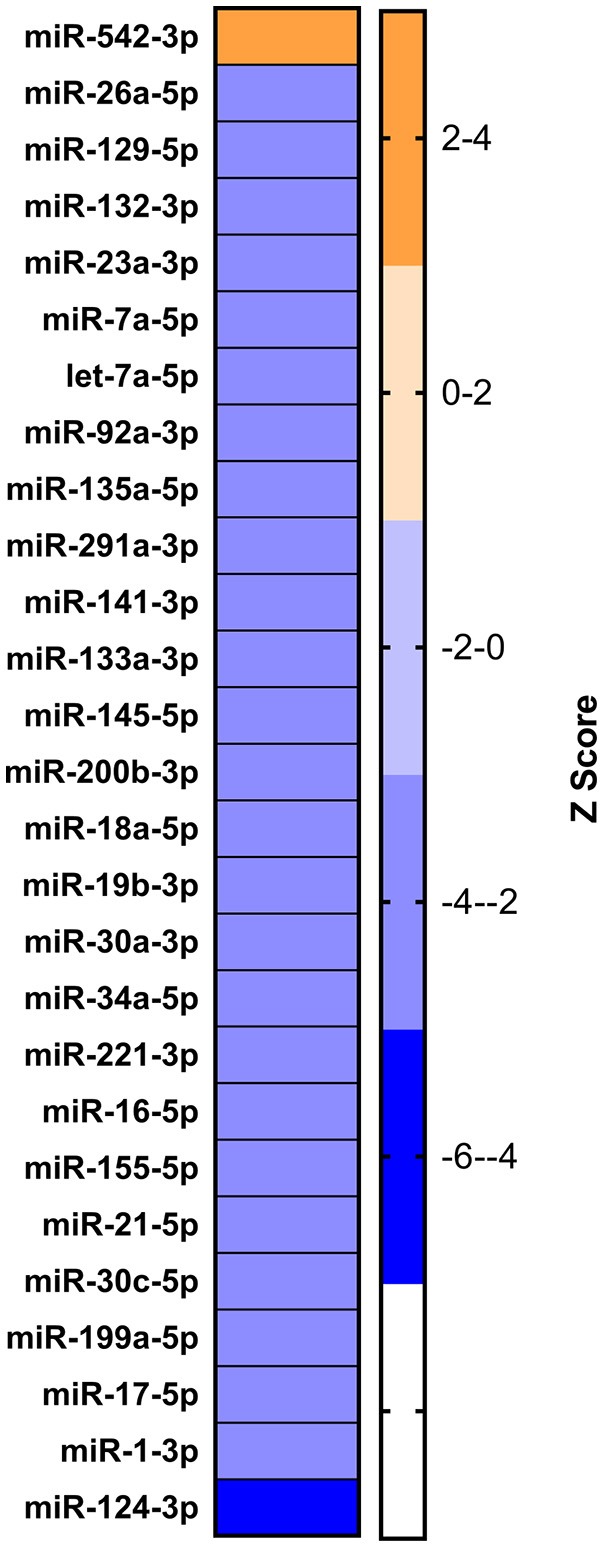
Identification of dysregulated miRNA from RNA sequencing data. IPA analysis to identify the upstream regulators governing the differentially expressed genes yielded 27 miRNAs.

On analyzing RNA-seq data (from the lab) of the 9 dpi PBMCs, 5,150 differentially expressed genes (DEGs) were identified in PPRV infected goats. These genes were further subjected to IPA using various modules based on knowledge database to predict the biological function of DEGs, the role of the molecules in various disease processes, upstream regulators (transcription factors, miRNAs, and drugs) that regulate the function of the downstream target genes and possible interactions among them. In the present study, we concentrated only on those miRNAs, which act as upstream regulators for DEGs. Twenty-seven miRNAs were identified regulating these DEGs. Of the 27 identified miRNAs, 26 were inhibited (z score < −2) and only 1 was activated (z score >2) (Figure 4). Further, out of these 27, only four miRNAs viz miR-129, miR-21-5p, Let-7a, and miR-200 were found be differentially expressed in the miRNA-seq data. miR-21-5p was found highly downregulated in miRNA-seq data (Table 2), inhibited in RNA-seq data and involved in regulation of various immune response genes. This miR-21-5p was further selected for annotation and functional validation.

### Validation of miR-21-5p by qRT-PCR

**Figure 5:**
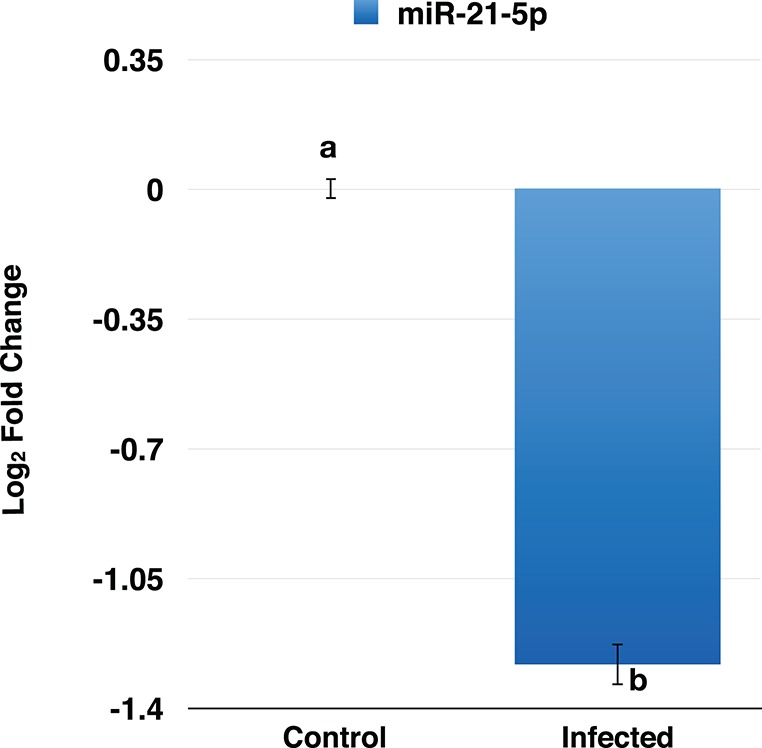
RT-qPCR analysis of miR-21-5p in PPRV infected goats PBMCs. The miR-21-5p was found to be downregulated in PPRV infected goat PBMCs.

To confirm the downregulation of miR-21-5p, qRT-PCR was used to validate its expression in PPRV infected goat PBMCs. This miRNA was found to be downregulated and was in concordance with the miRNA-seq results (Figure 5).

### Prediction of miRNA-21-5p targets, gene ontology analysis, and target selection for functional validation

**Figure 6:**
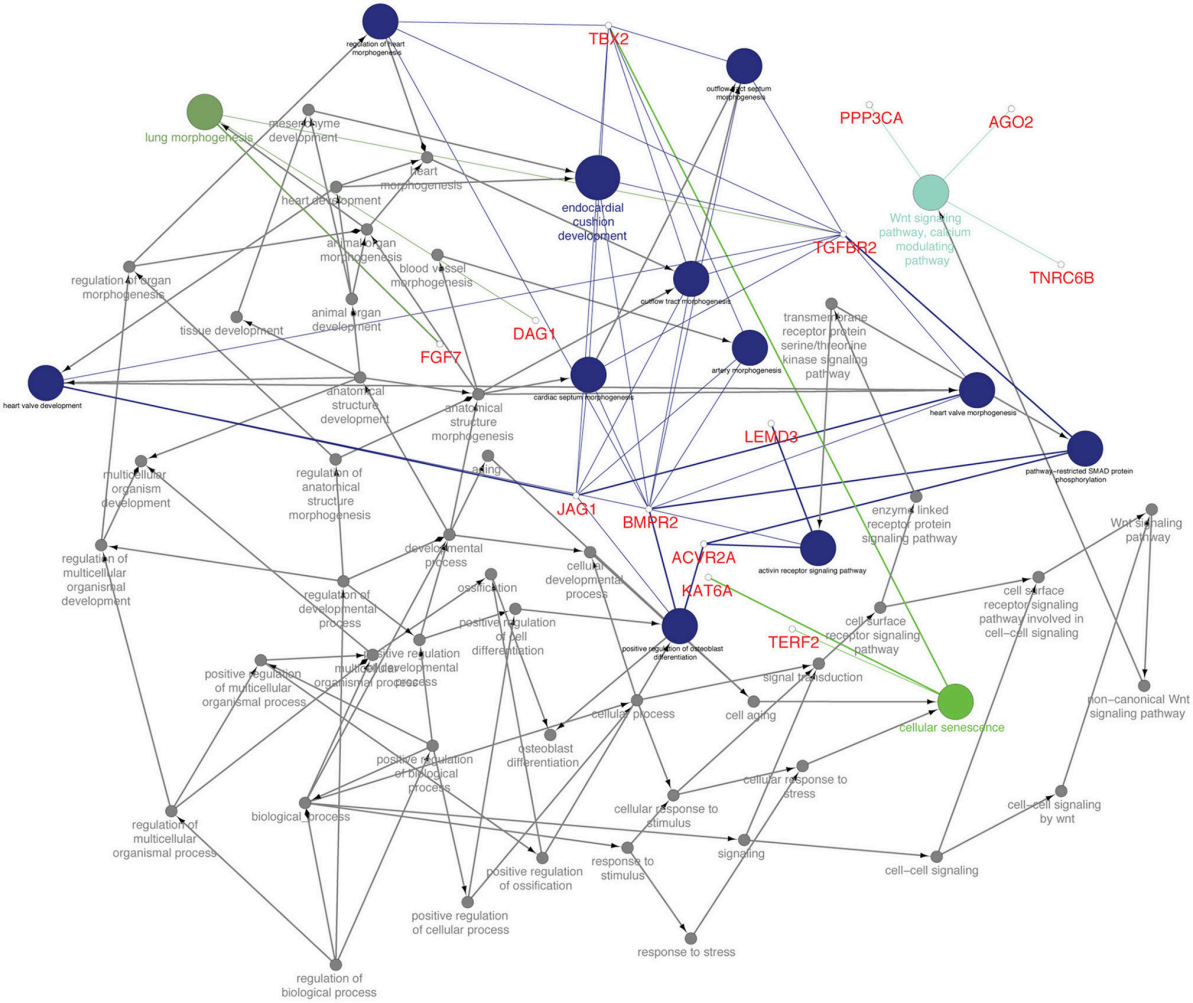
Functionally annotation of miR-21-5p based on the upregulated target genes (45). Gene ontology was visualized in these genes visualized in ClueGo (ver. 2.3.3) + CluePedia (ver. 1.3.3) plugin of Cytoscape (ver. 3.2.1).

From the TargetScan data, 356 genes were found to be governed by miR-21-5p, out of which 66 proteins were found to be upregulated (since miR-21-5p was downregulated, the study was concentrated only on upregulated proteins) in the proteomics data. These target proteins on GO analysis were enriched in Wnt signaling pathway, cell surface receptor signaling pathway, pathway-restricted SMAD phosphorylation, morphogenesis processes, positive regulation of cellular processes, multicellular organismal development, etc., (Figure 6). *TGFBR2* gene, which is connected to most of the GO terms viz pathway-restricted SMAD phosphorylation, activin receptor signaling pathway, Wnt signaling pathway, morphogenesis of lung and heart and blood vessels, and osteoclast differentiation was selected as the target of miR-21-5p. *TGFBR2* was also found to be governed by 21 identified immune regulating DEmiRNAs (Table 2 and Table S7).

### miRNA-protein regulatory network of miR-21-5p

**Figure 7:**
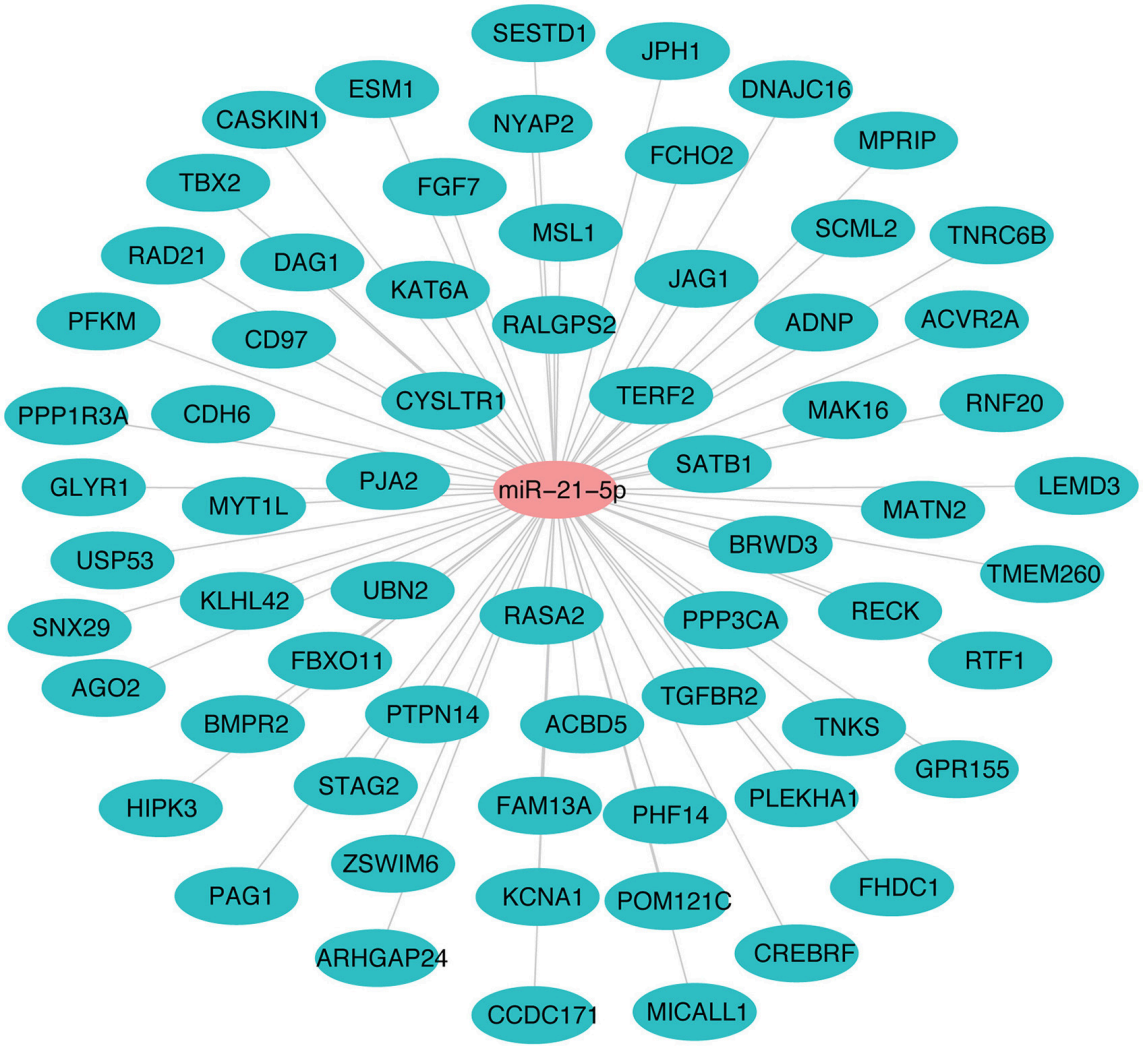
miRNA-protein interaction network of target proteins upregulated due to downregulation of miR-21-5p. Student's t-test was done in JMP9 (SAS Institute Inc, Cary, USA) to test the significance of difference and difference between groups was considered significant at *P* ≤ 0.05.

The miR-21-5p and 66 upregulated proteins interacting with it are represented in a network (Figure 7). Among the 66, 15 proteins (ACBD5, ADNP, CD97, CDH6, CREBRF, CYSLTR1, DNAJC16, FBXO11, HIPK3, JAG1, KAT6A, PAG1, PJA2, RAD21, *TGFBR2*) were found to be involved in immune response processes (Table 3). This suggested the involvement of miR-21-5p in the regulation of immune response in PPRV infected PBMCs.

**Table 3:** Immune-related functions of proteins upregulated due to downregulation of miR-21-5p.

**Protein symbol**	**Protein Name**	**Function**
ACBD5	Acyl-CoA binding domain containing 5	It acts as the peroxisome receptor for degradation of damaged peroxisomes and proteins (46). Mutation in the gene is associated with Thrombocytopenia (47).
ADNP	Activity-dependent neuroprotector homeobox	It is a neuroprotective molecule (45).
CD97	Adhesion G protein-coupled receptor E5	Leukocyte receptor involved in wide range of functions including cell adhesion and migration (48, 49).
CDH6	Cadherin 6	Role in Homophilic cell adhesion (50).
CREBRF	CREB3 regulatory factor	It assists unfolded protein response during endoplasmic reticulum stress (51).
CYSLTR1	Cysteine leukotriene receptor 1	It is involved in stimulating the activity of mast cells, eosinophil, dendritic cells and neutrophils (52, 53).
DNAJC16	DnaJ heat shock protein family (Hsp40) member C16	It belongs to DnaJ/Hsp40 family that acts as co-chaperone for Hsp70 proteins mediating folding of substrates in cytosol during cell stress (54).
FBXO11	F-box protein 11	It regulates Pr-Set7/Set8-Mediated Cellular Migration (55).
HIPK3	Homeodomain-interacting protein kinase 3	It promotes Resistance to Fas-mediated Apoptosis in DU 145 Prostate Carcinoma Cells (56).
JAG1	Jagged 1	It binds with the CD46 receptor and mediates induction of interferon-γ (IFN-γ)-secreting effector T helper type 1 (TH1) cells and their subsequent switch into interleukin 10 (IL-10)-producing regulatory T cells (57).
KAT6A	Lysine acetyltransferase 6A	Increases effector-like memory CD8+ T cells and cell surface CD8 and TCR levels (58).
PAG1	Phosphoprotein membrane anchor with glycosphingolipid microdomains 1	The complex of PAG with tyrosine kinase (Csk) transmits negative regulatory signals and thus may help to keep resting T cells in a quiescent state (59).
PJA2	Praja ring finger ubiquitin ligase 2	It regulates transcription of HIV Virus by degrading Tat (60).
RAD21	RAD21 cohesin complex component	Plays a role in apoptosis, via its cleavage by caspase-3/CASP3 or caspase-7/CASP7 during early steps of apoptosis (61).

### miR-21-5p and miR-484 were functionally validated using dual-luciferase reporter assay

**Figure 8:**
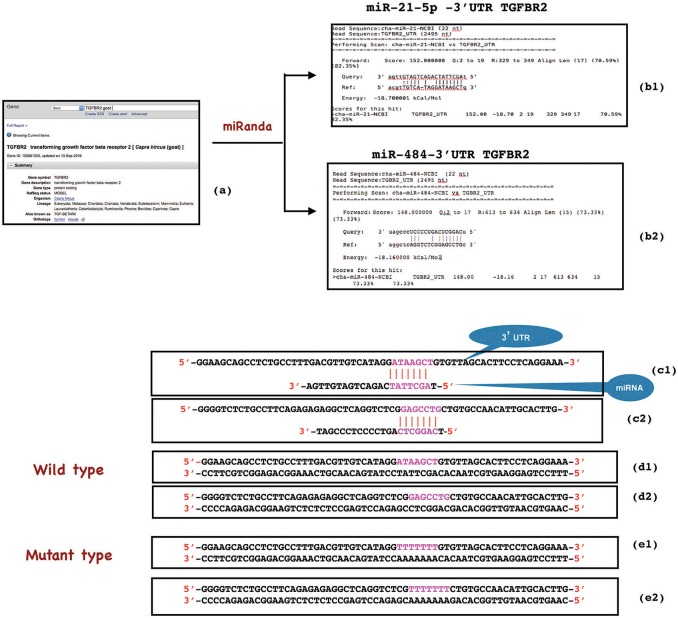
Construction of wild type and mutant type sequences flanking the miRNA target sequence. 3'UTR target site of *TGFBR2* gene was retrieved from NCBI **(a)** and analyzed in miRanda **(b1,b2)**. The complementary binding (in red), ΔG value (−18.70 and−18.16) and total score value (152 and 148) indicated the strength of interaction between miR-21-5p and *TGFBR2* (329-349 3'UTR), and miR-484 and *TGFBR2* (613-634 3'UTR) respectively **(b1, c1, b2, c2)**. While wild type oligonucleotide (62 bp) was constructed from 3'UTR of *TGFBR2* mRNA flanking the miRNA target site **(d1, d2)**, the mutant was created by replacing the target site either with poly A or poly T sequence (red) **(e1, e2)**.

**Figure 9:**
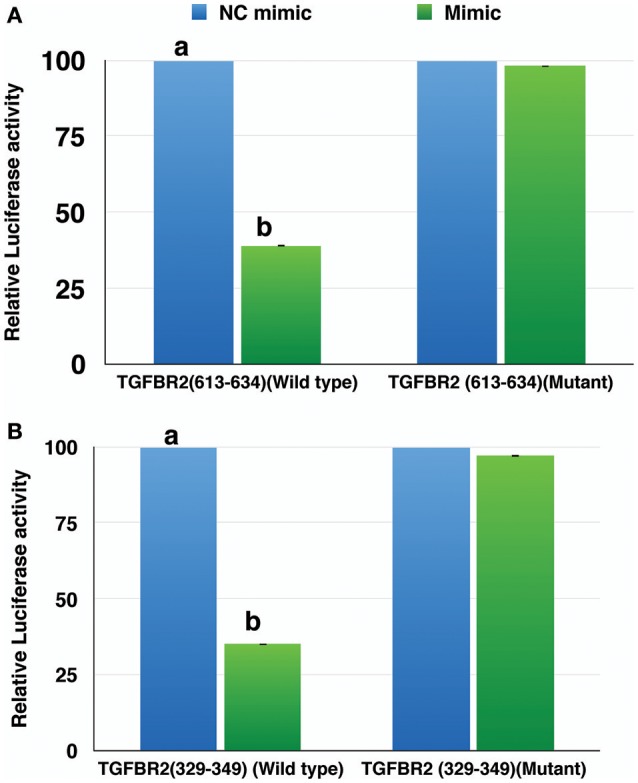
Relative luciferase activity in wild type and mutant of *TGFBR2* gene. The decrease in luciferase activity in the wild type indicated specific binding of miR-484 **(A)** and miR-21-5p **(B)** to their target site. Student's *t*-test was done in JMP9 (SAS Institute Inc, Cary, USA) to test the significance of difference and differences between groups were considered significant at *P* ≤ 0.05.

The miR-21-5p and miR-484 sequences were found to be complementary to sequences from 329-349 and 613-634 at 3′ UTR of *TGFBR2* gene, respectively. Further, the strength of interaction between the target site on *TGFBR2* for miR-21-5p as evaluated on the basis of ΔG value and total score value was−18.70 kCal/Mol and 152, respectively. The parameters of the interaction for the miR-484 and its target site at 3′ UTR of *TGFBR2* were−18.16 kCal/Mol and 148 (Figure 8). The wild-type and mutated type target sequences of *TGFBR2* were cloned into XbaI site of the vector pGL4.13. The transfection results showed complementary binding between miRNA mimics and their specific target sites expressed in the form of decreased expression of the luciferase gene in case of wild-type construct and increased expression in case of mutant type (Figure 9). The overview of abstract form of results is given in Data Sheet 1.

## Discussion

In an attempt to explore the role of microRNAs in modulating the host immune response against PPRV infection, we studied the differential expression of miRNAs in PPRV infected PBMCs, evaluated the influence of these DEmiRNAs on immune response processes from the proteomics data and functionally validated two miRNAs through Dual-Luciferase Reporter Assay.

The miRNA-seq data was analyzed in CLC genomics^3^ The analysis tools in CLC Genomics Workbench are designed to facilitate trimming of reads, and counting and annotation of the resulting tags using miRBase in general and microRNA of reference organism in particular. Functional analyses of miRNAs or miRNA high-throughput datasets commonly use the Gene Ontology annotations associated with the genes or gene products that the miRNAs are predicted to regulate. Therefore, it is critical to identify targets for understanding their biological function and molecular mechanism (62). TargetScan, an online tool allows the user to extract target genes against broadly conserved or poorly conserved miRNA families across several species or target miRNAs against a particular gene. Thus, it is imperative to identify proteins that are regulated by miRNAs.

The miRNA-protein network analysis suggests that one miRNA could participate in several biological processes by targeting different mRNAs, and one biological process could be influenced by multiple miRNAs (2). The DEmiRNAs identified were found to govern 98 genes that regulate several immune response pathways. miR-664, miR-2311, miR-484, miR-2440, miR-574, miR-210, miR-21-5p, miR-2897, miR-3533, and miR-30 were the top 10 miRNAs governing the immune response processes. miR-664 has been demonstrated to be upregulated during influenza A infection of A549 cells and was found to have proviral activity (63). Similarly, overexpression of miR-484 has been found to inhibit Dengue viral infection *in-vitro* (64). These miRNAs—miR-664 and miR-484 were found to be upregulated in our study indicating their possible role in the immune response in PPRV infected PBMCs. miR-210 has been reported to inhibit apoptosis (65). This miR-210 was downregulated in PPRV infected PBMCs thus promoting apoptosis in animals that succumbed to the disease. PPRV has been reported to cause apoptosis in PBMCs (66). miR-21-5p has been found to decrease the sensitivity of the cell to the antiviral activity of IFNs, decrease the production of the Th1 cytokine IFN γ and inactivate the T-cell (67), thus facilitating viral replication. In our previous study, IFN γ has been found to increase in PBMCs infected with PPRV infection (35). So, the downregulation of miR-21-5p could be a contributing factor for the increase in IFN γ level during PPRV infection. miR-30b plays an inhibitory role in antigen processing and presentation by primary macrophages and dendritic cells (68) and suppresses TLR/MyD88 activation and cytokine expression in THP-1 cells during MTB H37Rv infection (69). The downregulation of miR-21-5p and miR-30 in our study is indicative of strong host response to PPRV infection.

To investigate the role of immune regulating DEmiRNAs in regulating immune response genes in goat, we choose to confirm the binding between miRNAs and a common gene to evaluate the effect of the interaction. Here, we selected miR-21-5p for functional validation vis-à-vis *TGFBR2* as a target gene. *TGFBR2* along with TGFBR1 transduces signals of cytokines like TGFB1, TGFB2, and TGFB3 from the cell surface to cytoplasm (70). TGFB signaling pathway plays an important role not only in tissue development and morphogenesis (71, 72) but also in wound healing (73). In this study, *TGFBR2* was found to be regulated by 21 identified DEmiRNAs including most of the top 10 immune regulating miRNAs. We identified *TGFBR2* to be connected to various development processes like lung morphogenesis and cardiovascular system in GO analysis, suggesting its role in restoring host physiology under PPRV infection. Further, the development, differentiation, and tolerance of T cells and homeostasis of T and B cells are regulated separately by TGFB signaling pathway and Wnt signaling pathway (74–76). Activin receptor signaling pathway shares the SMAD proteins with TGFB signaling pathway (77, 78) and plays a crucial role in inflammation (79). The involvement of *TGFBR2* in TGFB signaling pathway and its direct association with Activin receptor signaling pathway and indirect association with Wnt signaling pathway, as predicted in the GO analysis in this study, highlights how TGFBR2 regulates immune response under PPRV infection. Moreover, miR-484 was selected at random from the top 10 immune regulating DEmiRNAs for functional validation against *TGFBR2* to provide further evidence of interaction between miRs and genes.

Genetic reporter systems provide efficient means of studying the regulation of eukaryotic gene expression by exploiting the “biochemical requirements” for luminescence of reporter molecules (80). In Dual Luciferase Reporter **(**DLR) Assay, the target sites of the *TGFBR2* for miR-21-5p and for miR-484 were cloned into pGL4.13 vector having luciferase reporter. The interaction between the target mRNA and the specific miRNA mimic lead to an inevitable knockdown in expression of the target mRNA. The ligation of the target site (3'UTR) of *TGFBR2* to the luciferase reporter gene in the vector pGL4.13 prevent the luciferase gene from getting translated whenever miRNA-21-5p and miR-484 mimics were cotransfected with the vector. Hence, the significant reduction in expression of luciferase activity in wild-type *TGFBR2* in comparison to mutant type *TGFBR2* was observed.

In this study, we identified miRNAs that are instrumental in regulating immune response to PPRV infection following integrative analysis of miRNA-seq data and proteome profiling data.

## Author contributions

RS, BPM, and RG conceived and designed the research. KR and DM performed the vaccine potency experiment. YS and RG maintained the server for analysis. AK, ARS, SW, and SS conducted the wet lab work. AK, ARS, RK, AP, and RG analyzed the data. RK, WM, RG, APS, PM, and BM helped in manuscript drafting and editing. RS, BPM, and RG proofread the manuscript.

### Conflict of interest statement

The authors declare that the research was conducted in the absence of any commercial or financial relationships that could be construed as a potential conflict of interest.

## Footnotes

^1^http://www.targetscan.org/vert_71/.

^2^https://www.qiagenbioinformatics.com/products/ingenuity-pathway-analysis/

^3^https://www.qiagenbioinformatics.com/products/clc-genomics-workbench/.

## Supplementary material

Table S1 Summary of miRNA sequencing data.

Table S2 Proteome data.

Table S3 Gene Symbol of proteins identified in GO terms pertinent to immune response.

Table S4 Total immune response genes governed by various upregulated miRNAs.

Table S5 Total immune response genes governed by various downregulated miRNAs.

Table S6 Comaprsion of DEmiRNA from PPRV infected PBMCs with DEmiRNA from PPRV infected lung and spleen.

Table S7 DEmiRNAs that regulates the expression of *TGFBR2*.

Data Sheet 1 Overview of results.
