# Supplementary material for: Dysregulated miRNAome and Proteome of PPRV Infected Goat PBMCs Reveal a Coordinated Immune Response
Source: Front Immunol. 2018 Nov 21;9:2631. doi: 10.3389/fimmu.2018.02631 (PMC6262310; doi:10.3389/fimmu.2018.02631)
Supplement: Data Sheet 1 — Overview of results. [file Data_Sheet_1.PDF]

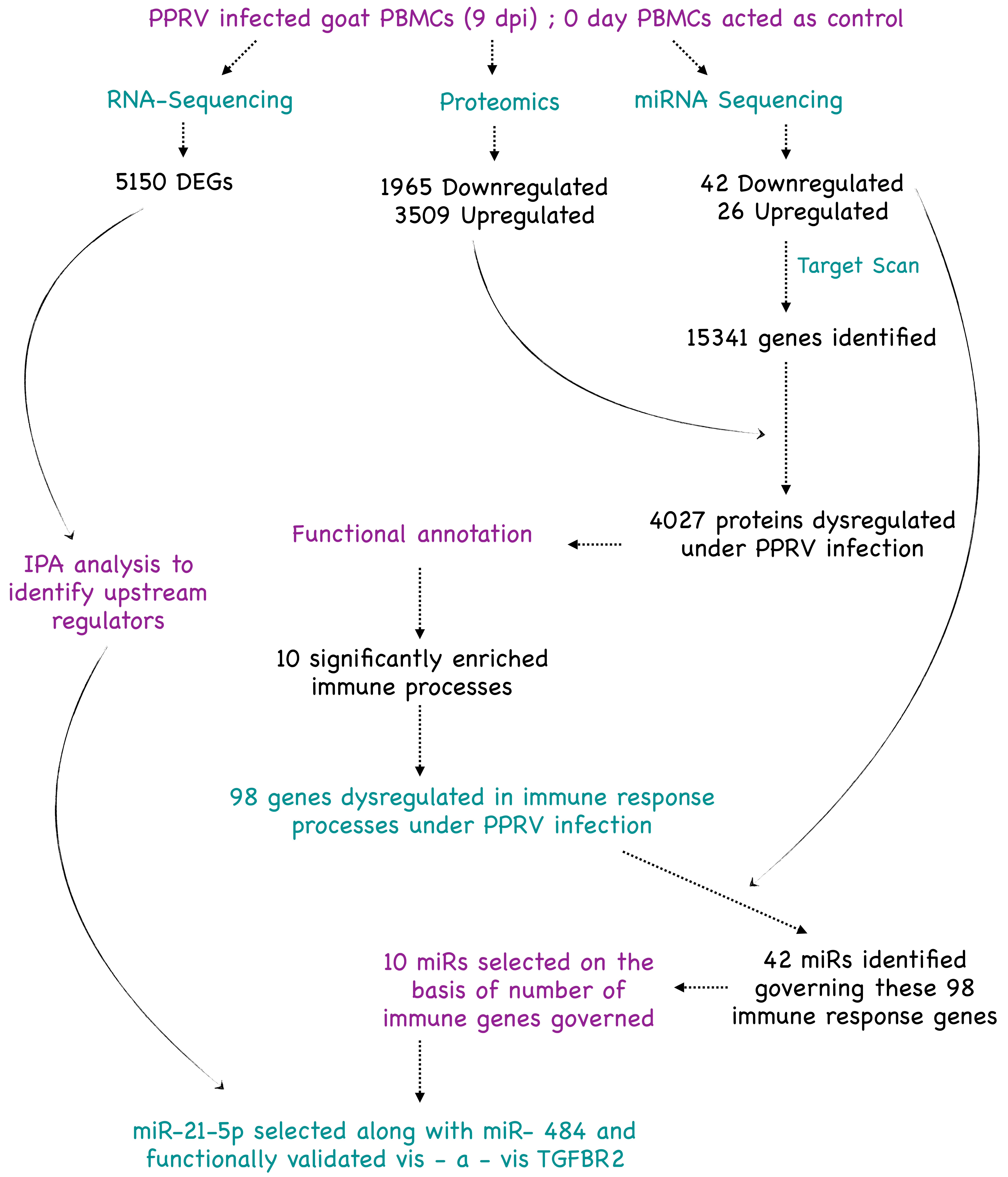
PPRV infected goat PBMCs (9 dpi) ; 0 day PBMCs acted as control
RNA-Sequencing
Proteomics
miRNA Sequencing
5150 DEGs
1965 Downregulated
3509 Upregulated
42 Downregulated
26 Upregulated
Target Scan
15341 genes identified
4027 proteins dysregulated
under PPRV infection
Functional annotation
IPA analysis to
identify upstream
regulators
10 significantly enriched
immune processes
98 genes dysregulated in immune response
processes under PPRV infection
42 miRs identified
governing these 98
immune response genes
10 miRs selected on the
basis of number of
immune genes governed
miR-21-5p selected along with miR- 484 and
functionally validated vis - a - vis TGFBR2
